# Better osteoarthritis care requires prevention and stratification

**DOI:** 10.1016/j.mmr.2026.100073

**Published:** 2026-10-06

**Authors:** David J. Hunter, Benjamin G. Faber, Kai Fu

**Affiliations:** Sydney Musculoskeletal Health, Kolling Institute, Faculty of Medicine and Health, the University of Sydney, Sydney, NSW 2006, Australia; Department of Rheumatology, Royal North Shore Hospital, Northern Sydney Local Health District, St Leonards, NSW 2065, Australia; Sydney Musculoskeletal Health, Kolling Institute, Faculty of Medicine and Health, the University of Sydney, Sydney, NSW 2006, Australia; Musculoskeletal Research Unit, University of Bristol, Bristol, BS10 5NB, UK; MRC Integrative Epidemiology Unit, University of Bristol, Bristol, BS1 5DS, UK; Department of Orthopedic Surgery, Shanghai Sixth People’s Hospital, Shanghai Jiao Tong University School of Medicine, Shanghai 200233, China

**Keywords:** Osteoarthritis, Disease modification, Precision medicine, Prevention, Implementation

Osteoarthritis (OA) is among the most common causes of pain and disability worldwide. An estimated 595 million people were living with OA in 2020, and the number affected at major joint sites is projected to rise dramatically by 2050 [1]. The scale is enormous. Yet, despite decades of scientific progress, the everyday experience of many people with OA has changed remarkably little and is characterized by pain, functional loss, fragmented care and, for some, joint replacement. No disease-modifying OA drug (DMOAD) has yet secured regulatory approval [2]. That matters, and it should prompt a more uncomfortable question: are we simply waiting for the right molecule, or are we still framing the disease, and therefore its solutions, too narrowly?

For much of its history, OA has been conceptualized primarily as a disease of cartilage. That model is no longer tenable. OA is a heterogeneous disorder of the whole joint, involving cartilage, subchondral bone, synovium, meniscus, ligaments, muscle, and neural pathways. The biology is more complex. Aging, injury, abnormal mechanical loading, obesity, metabolic dysfunction and inflammation shape the development and expression of OA, often in different combinations across the life course [3]. Pain adds another layer. It is the symptom that matters most to patients, yet it is not a direct read-out of cartilage loss and may arise from several joint tissues, altered peripheral nociception and changes in central pain processing and psychosocial influences [4]. In practice, that disconnect is familiar. The next advance will require us to think beyond cartilage and, increasingly, beyond the joint itself.

The heterogeneity has profound implications for therapeutic development. The “average patient with OA” is, in many respects, a statistical fiction. Two people with apparently similar radiographic disease may have different dominant mechanisms of pain and progression and very different responses to the same treatment [5], [6]. Trials that enroll broadly defined OA populations and seek modest average effects from a single intervention are therefore biologically inefficient, even if statistically elegant. The important question is no longer simply whether a treatment works in OA. In whom does it work? At what stage? Against which mechanism? Moving from clinical phenotypes towards biologically informed endotypes and actionable theratypes should become a core organizing principle for OA therapeutic development. Classification systems often become more complicated before they become useful. Still, the direction is clear.

We must also broaden what we mean by disease modification. Traditionally, disease-modifying therapy has meant slowing structural deterioration once OA is already established. That might be too late. Some of the strongest determinants of OA, particularly joint injury and obesity, act years before severe symptoms arise. Preserving muscle, maintaining physical activity and addressing excess adiposity and metabolic risk are not peripheral “lifestyle” issues; within a life-course model of OA, they may be genuinely disease-modifying [7]. This is not simply semantics. Preventing persistent pain, loss of participation and progression to joint replacement should matter at least as much as slowing a radiographic measurement by a fraction of a millimetre (Fig. 1). Perhaps the clearest illustration comes from outside conventional OA drug discovery. In the STEP 9 trial, semaglutide produced substantially greater weight loss than placebo in people with obesity and symptomatic knee OA (13.7% versus 3.2%), alongside substantially greater improvement in knee pain [8]. The precise contributions of reduced mechanical loading, systemic metabolic effects, inflammation, and other mechanisms remain uncertain. They probably overlap. The conceptual lesson is more important whereby targeting a specific mechanism (weight loss) at an enriched OA subgroup (obese individuals with knee OA) yielded success. In a single trial, obesity medicine delivered one of the most striking recent therapeutic advances in symptomatic knee OA, rather than a cartilage-targeted OA programme.

**Fig. 1:**
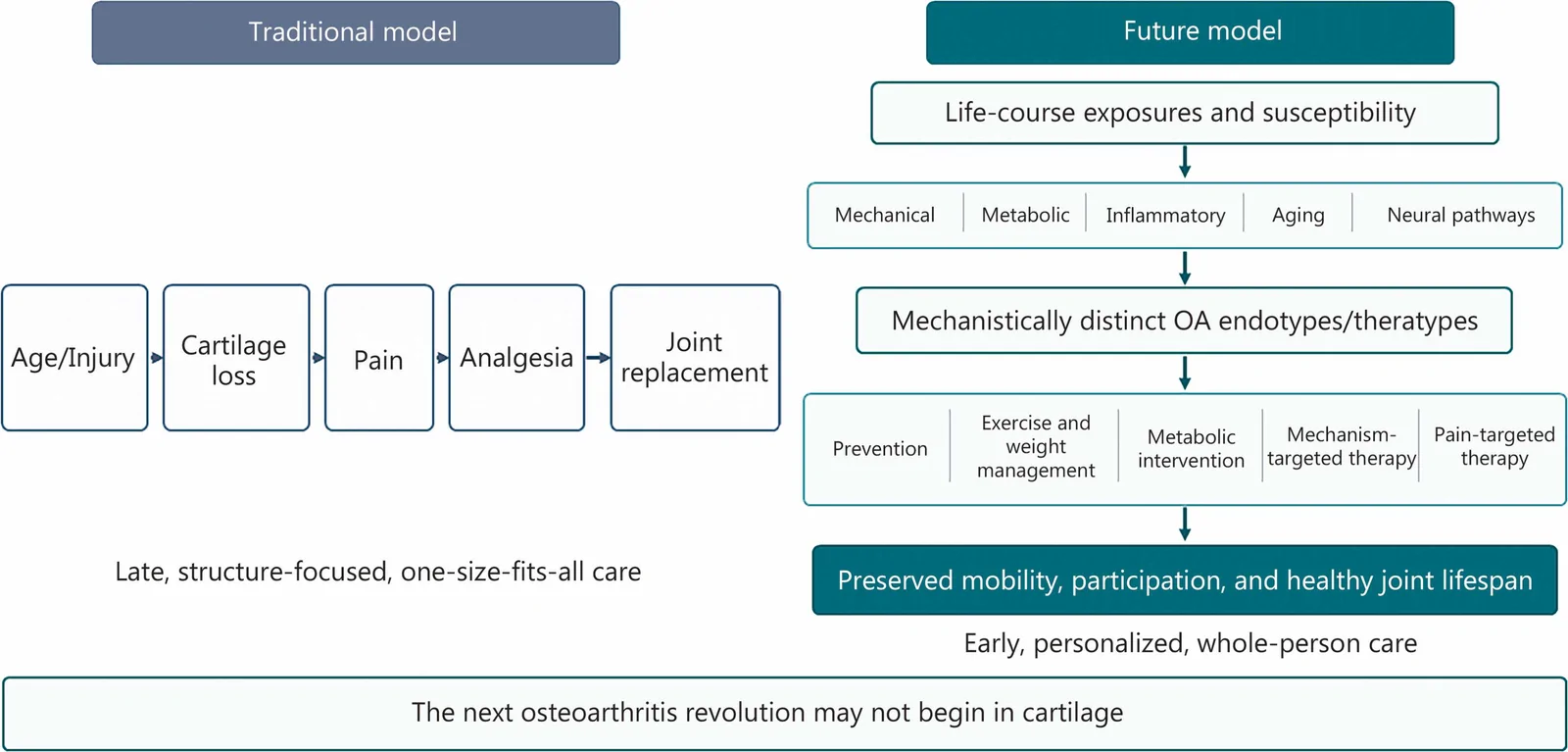
Rethinking osteoarthritis: from a cartilage-centric to a whole-person model. OA. Osteoarthritis.

That lesson should shift where the field looks next. The next major advance may come from obesity medicine, neurobiology, immunometabolism, regenerative biology, or interventions that modify muscle and bone. Perhaps several of these mechanistic targets all at once. OA should no longer be treated as an isolated musculoskeletal condition because its biology intersects with aging and multimorbidity, while its consequences extend to physical inactivity, work participation, loss of independence and healthy longevity. Cross-disciplinary discovery may prove more productive than an ever more detailed search within traditional OA silos.

There is another uncomfortable reality. We already have treatments that improve pain and function, including education, exercise, physical activity, and weight management, yet their delivery in routine care remains inconsistent. A health system cannot credibly describe OA as “untreatable” while failing to provide the treatments known to help. Our health care systems continue to support low-value care interventions including opioids and arthroscopy while limiting or not reimbursing care that we know works, including joint replacements where appropriate.

Implementation science, coordinated models of primary care, scalable digital support and better access to exercise and weight-management services deserve the same seriousness as molecular discovery. This is less glamorous. It may matter more at the population scale. A highly effective intervention that reaches few eligible patients can have less population impact than a moderately effective intervention delivered reliably to millions.

The future of OA therefore requires several transitions: from cartilage to the whole person; from established disease to life-course prevention; from the average patient to the mechanistically defined theratype; from OA-specific silos to cross-disciplinary therapeutics; and from discovery alone to reliable delivery. We should continue searching for molecular treatments that can alter joint biology. Emerging targeted therapies may finally change what is possible. But that cannot be the whole strategy. The transformation of OA care may arrive partly in a syringe or tablet, partly through metabolic or behavioural treatment, partly through prevention, and partly through something less dramatic: consistently delivering effective care to the people who need it.

The next OA revolution may not begin in cartilage. And the next breakthrough may not be an OA drug.

Abbreviations

OA: Osteoarthritis

## Ethics approval and consent to participate

Not applicable.

## Funding

This work was supported by the Arthritis Australia and an NHMRC Investigator Grant Leadership 2 of Australia (#1194737 to DJH), and a Wellcome Trust Early Career Award of the United Kingdom (316390/Z/24/Z to BGF).

## Competing interests

DJH is the editor of the osteoarthritis section for UpToDate and co-Editor-in-Chief of Osteoarthritis and Cartilage. DJH provides consulting advice on scientific advisory boards for Haleon, Naia Health, Novartis, Tissuegene, Lilly, Doron, Sanofi, and Formation Bio. BGF has provided consulting for Relation Therapeutics and received honoraria from UCB and Calico Life Sciences.

## Data Availability

Not applicable.
